# Histone H1 Differentially Inhibits DNA Bending by Reduced and Oxidized HMGB1 Protein

**DOI:** 10.1371/journal.pone.0138774

**Published:** 2015-09-25

**Authors:** Michal Štros, Eva Polanská, Martin Kučírek, Šárka Pospíšilová

**Affiliations:** 1 Laboratory of Analysis of Chromosomal Proteins, Institute of Biophysics, Academy of Science of the Czech Republic, Brno, Czech Republic; 2 Central European Institute of Technology (CEITEC) Center of Molecular Medicine, Masaryk University, Brno, Czech Republic; National University of Singapore, SINGAPORE

## Abstract

HMGB1 protein and linker histone H1 have overlapping binding sites in the nucleosome. HMGB1 has been implicated in many DNA-dependent processes in chromatin involving binding of specific proteins, including transcription factors, to DNA sites pre-bent by HMGB1. HMGB1 can also act as an extracellular signaling molecule by promoting inflammation, tumor growth a metastasis. Many of the intra- and extracellular functions of HMGB1 depend on redox-sensitive cysteine residues of the protein. Here we report that mild oxidization of HMGB1 (and much less mutation of cysteines involved in disulphide bond formation) can severely compromise the functioning of the protein as a DNA chaperone by inhibiting its ability to unwind or bend DNA. Histone H1 (via the highly basic C-terminal domain) significantly inhibits DNA bending by the full-length HMGB1, and the inhibition is further enhanced upon oxidization of HMGB1. Interestingly, DNA bending by HMGB1 lacking the acidic C-tail (HMGB1ΔC) is much less affected by histone H1, *but* oxidization rendered DNA bending by HMGB1ΔC and HMGB1 equally prone for inhibition by histone H1. Possible consequences of histone H1-mediated inhibition of DNA bending by HMGB1 of different redox state for the functioning of chromatin are discussed.

## Introduction

HMGB1 is an “architectural” chromatin-associated protein, a member of the High Mobility Group (HMG^2^) superfamily. The protein has been implicated in many DNA-dependent processes in chromatin, as well as in cell signaling, promotion of tumor growth and metastasis, reviewed in [1–5]. Association of HMGB1 is not confined to specific sites in chromatin but it is rather highly dynamic, and the protein can scan the potential binding sites and move from one chromatin site to another in a “hit and run” fashion [6]. Reports from several laboratories provided evidence that many of the intracellular and extracellular functions of HMGB1 depend on redox-sensitive cysteine residues of the protein, reviewed in [7]. All-thiol (reduced) HMGB1 acts as a chemoattractant, but a disulphide bond within (oxidized) HMGB1 can turn it into a pro-inflammatory cytokine [8] and refs. therein. Changes in the redox state of HMGB1 can also impair the binding affinity of the protein to bent (cisplatin-modified) or superhelical DNA [9–12], as well as the ability of the protein to bend DNA [13].

HMGB1 contains two unique DNA-binding domains, the HMG-boxes (referred to as domains A and B), linked by a short basic linker, reviewed in [4]. The HMG-boxes can bind non-B-type DNA structures (bent, kinked and unwound) with high affinity, and also distort DNA by bending/looping and unwinding, reviewed in [1–4,14]. DNA binding properties of the HMGB1 domains A and B are modulated by the acidic C-tail, consisting of an unbroken run of 30 Glu/Asp residues, reviewed in [4]. The acidic tail of HMGB1 is involved in modulation of interactions of the HMG-boxes of HMGB1 with a plethora of cellular proteins, and it can also directly interact with some proteins, reviewed in [4].

Histones of H1 family are chromatin proteins, approximately 10–15 times more abundant than HMGB1, and bind with a stoichiometry of approximately one copy per nucleosome, reviewed in [4,15]. Histone H1 and HMGB1 are structurally unrelated proteins with overlapping binding sites in the nucleosome, suggesting that the presence of both proteins in the nucleosome is mutually exclusive, reviewed in [15]. While HMGB1 has been implicated in loosening of chromatin structure by DNA distortion and direct protein-protein interactions (augmenting binding of specific proteins to DNA), histones H1 are responsible for maintaining and stabilizing higher-order chromatin structure. Linker histones H1 have been shown to affect interactions of the HMG boxes of HMGB1 with DNA and chromatin-associated proteins, presumably via competing off the acidic C-tail [4,15–18].

In this study we demonstrate that oxidization of cysteine residues 22 and 44 of HMGB1 (and much less mutation of the same residues to alanine) inhibits the ability of the protein to bend or unwind DNA. We also report that linker histone H1 (*via* its highly basic C-terminus) can suppress the DNA bending potential of HMGB1. The extent of the inhibition depends on the redox state of HMGB1, and is further modulated by the acidic tail of HMGB1. Possible consequences of histone H1-mediated inhibition of DNA bending by HMGB1 for the functioning of chromatin are discussed.

## Materials and Methods

### Expression and purification of HMGB1

His-tagged or untagged (upon cleavage of the N-terminal GST-fusion protein) recombinant HMGB1 domains A or B, and their mutants were expressed in *E*. *coli* BL21(DE3) cells from either the pET (Novagene) vectors or the glutathione S-transferase (GST) fusion expression vector pGEX-4T1pGEX4T1 encoding rat HMGB1 cDNAs (the amino acid sequence of the rat HMGB1 protein is identical to that of the human protein). Untagged wild-type HMGB1 protein (residues 1–214), HMGB1(F37A), HMGB1(F102A/I112A), HMGB1(C22A/C44A) mutants, and HMGB1-ΔC(residues 1–185) or HMGB1-ΔC(F37A) were expressed from pETite-N-his-SUMO-constructs prepared by using “Expresso T7 SUMO Cloning and Expression System” (Lucigen). All mutations we introduced using and “Site-directed Mutagenesis Kit” (ThermoFisher). HMGB sequences and introduced mutations were verified by dideoxy-sequencing of final plasmid DNA constructs on both strands. Purified HMGB1 protein or HMGB1domain A were oxidized under mild conditions to promote disulfide bond formation as detailed in [12,13]. Briefly, proteins were dialyzed overnight against DB buffer (0.15 M NaCl, 20 mM Hepes pH 7.9, 1 mM PMSF) containing 5 μM CuCl_2_, followed by re-dialysis against DB buffer lacking CuCl_2_. In some experiments, the oxidized HMGB1 protein was fully reduced by treatment with 10 mM DTT at 30°C for 30 min [please notice that for EMSA experiments with p53 protein, < 0.1 mM DTT had to be included in the reaction buffer to ensure proper binding of p53 to DNA (4°C). No detectable reduction of the oxidized HMGB1 or domain A was observed under these conditions]. The concentrations of the reduced or oxidized proteins were determined from the SDS-PAGE gel by the Coomassie Brilliant Blue G-250 Protein Assay (Bio-Rad) using BSA as standard (reduced and oxidized HMGB1 have been pre-treated with 10 mM DTT at 30°C for 30 min prior to the assay).

### MALDI-TOF mass-spectrometry

Formation of disulphide bond between cysteine residues 22/44 within HMGB1 domain A was determined by MALDI-TOF mass-spectrometry. Briefly, proteins were subjected to trypsin digestion. The digests (1:l) were mixed with CHCA (α-cyano-4-hydroxycinnamic acid) matrix solution on the Anchor Chip target in a 2:1 ratio. The digests were analyzed by using an Ultraflex III MALDI-TOF mass spectrometer (Bruker Daltonik, Bremen, Germany). An external calibration procedure was employed, using a mixture of seven peptide standards (Bruker Daltonik). Peptide maps were acquired in reflectron positive mode (25 kV acceleration voltage) with 800 laser shots and peaks with 0.70–4.1 kDa mass range and minimum S/N 10 were picked out for MS/MS analysis employing LID-LIFT arrangement with 600 laser shots for each peptide. The Flex Analysis 3.4 and MS Biotools 3.2 (Bruker Daltonik) software were used for data processing. MASCOT 2.4 (MatrixScience, London, UK) search engine was used for evaluation of the MS and MS/MS data. Database searches were done against the NCBI protein database. A mass tolerance of 100 ppm was allowed during processing MALDI MS data for PMF and 0.6 Da during processing LID-LIFT data for MS/MS ion searches. The MS/MS data of peptides containing disulphide bridges were interpreted manually.

### Histone H1 and truncated forms

Recombinant mouse histone H1° (referred throughout the manuscript as histone H1) and the CTD-truncated mutants were expressed from plasmids pET-H1°-11d, pET-H1°-CΔ24-11d pET-H1°-CΔ48-11d, pET-H1°-CΔ72-11d and pET-H1°-CΔ97-11d, respectively (the plasmids were kindly provided by Jeffrey C. Hansen, Colorado State University, Fort Collins, USA). Recombinant mouse histone H1 and its truncated forms were expressed in *E*. *coli* BL21(DE3) cells and purified on the CM-Sephadex C-25 column as detailed in [19]. The obtained fractions were analyzed by SDS/15%-PAGE, and the fractions containing purified proteins were combined and dialyzed against buffer D (10 mM Tris pH 8.0, 1 mM EDTA and 50 mM NaCl). The concentrations of the purified proteins were determined from the SDS-PAGE gel by the Coomassie Brilliant Blue G-250 Protein Assay (Bio-Rad) using BSA as a standard [13].

### Preferential binding of HMGB1 to supercoiled DNA

Preferential binding of HMGB1 and mutants to supercoiled DNA was carried out essentially as previously described [20]. Briefly, 1:1:1 mixtures of negatively supercoiled plasmid pBR322, relaxed-closed circular pBR322 and linearized pBR322 (each of the DNA forms was 150 ng) were mixed in buffer A (50 mM NaCl, 20 mM Hepes pH 7.9, 4% sucrose) with increasing amounts of HMGB1 proteins in a final volume of 20 μL. After 20 min pre-incubation on ice, samples were loaded on 1% agarose gels in 0.5 x TBE and subjected to electrophoresis for 15–18 h at 3V/cm, followed by staining with 10 000-fold diluted GelRed (Biotium).

### DNA supercoiling

Effect of HMGB1, HMGB1 mutants or HMGB1ΔC on topoisomerase I-mediated DNA supercoiling was studied essentially as previously described [11]. Briefly, negatively supercoiled plasmid pBR322 DNA (final concentration ≈ 170 μg/mL) was relaxed in relaxation buffer (150 mM NaCl, 25 mM Tris-HCl, pH 7.8, 1 mM EDTA, and 20% glycerol) with wheat germ topoisomerase I (4 units/μg DNA; Promega) at 37°C for 90 min. The relaxed DNA was then diluted in 25 mM Tris-HCl, pH 7.8 and 1 mM EDTA to final concentration 50 mM NaCl, followed by addition of a second portion of the enzyme. The DNA mixture was then divided into several tubes, each containing ∼10 nM DNA, and then the HMGB1 proteins were added (as indicated in the legend to figures) to a final volume of 20 μL. The reactions were allowed to proceed for 1 h at 37°C after which 5 μL of the termination mix (5× TBE, 5% SDS, 15% sucrose, 0.1% bromphenol blue, 0.1% xylenecyanol, 1 μg/μL proteinase K) was added and the samples were subjected to further incubation at 37°C for 1 h. DNA topoisomers were then resolved on 1% agarose gels in 0.5× TBE buffer at ∼3 V/cm for 17 h. The gels were stained with GelRed (Biotium), followed by visualization of the DNA topoisomers under UV-illumination (254 nm).

### The ligase-mediated DNA circularization assay

The assays were performed as previously described [13,21,22]. Briefly, the ^32^P-labeled 123-bp (*Ava*I ends) or 66-bp (*Nde*I ends) DNA duplexes (~1 nM) were ligated by T4 DNA ligase (0.05 units, Takara) in the absence or presence of HMGB1 (see the corresponding Figures) at 30^°^C for 40min. In some experiments, HMGB1 was pre-incubated with histone H1 or truncated forms of H1 (for exact concentrations of H1 *see* the Legends to Figures) for 20 min before addition of DNA ligase. Termination of ligation and treatment of samples with Proteinase K was performed as previously described [21]. Some of the samples were digested (prior to Proteinase K treatment) with 4–20 units of exonuclease III (Promega) at 37^°^C for 30 min. Deproteinised DNA samples were then resolved on pre-run 5% polyacrylamide gels in 0.5xTBE buffer (250 V for 4 h at 4^°^C), and DNA was visualized and quantified from dried gels on PhosphorImager Typhoon SLA9000 (GE).

## Results

### Oxidization of HMGB1 protein

**Fig 1:**
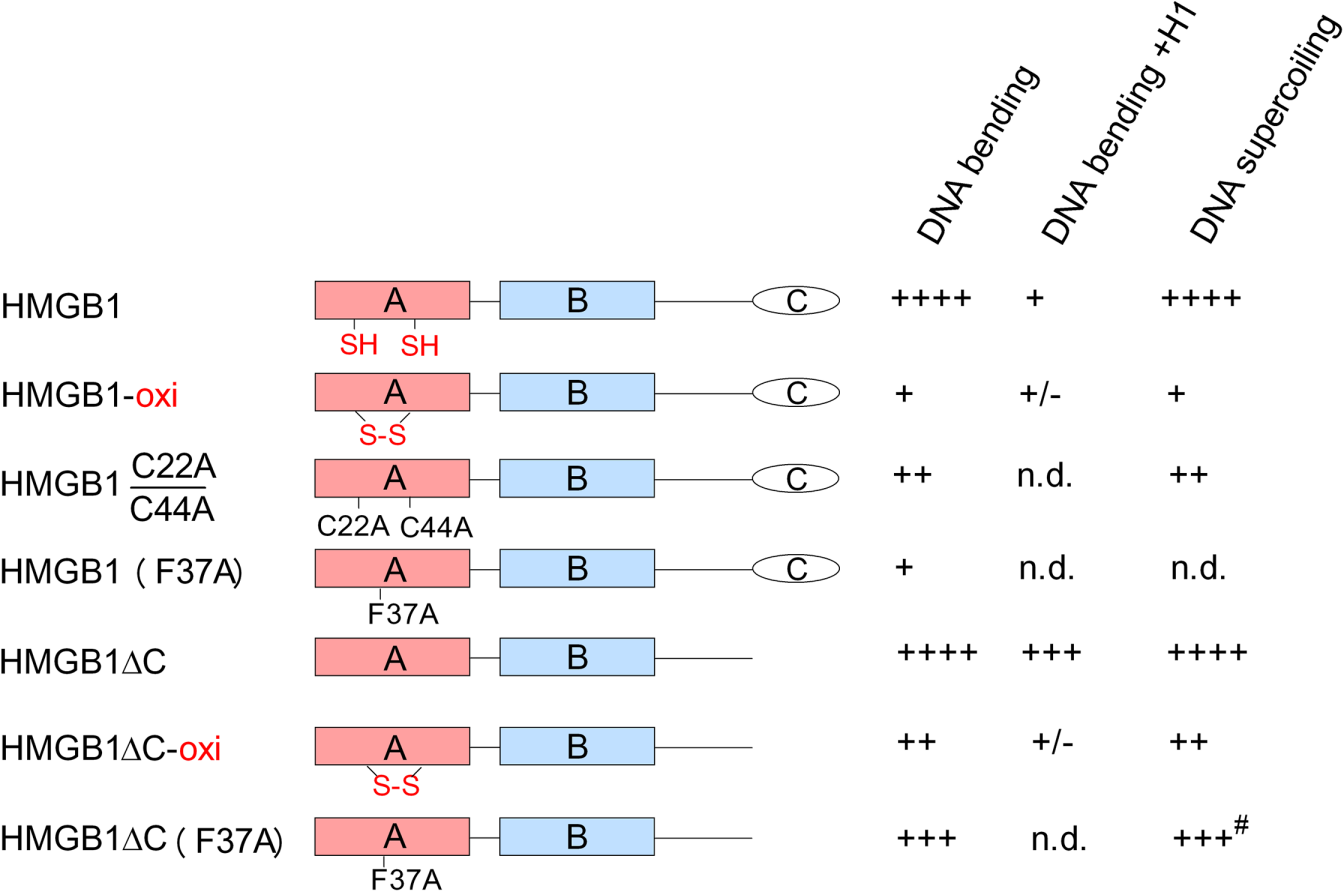
HMGB1 and peptides. (*top*) HMGB1 organization (amino acid residues 1–214; A, domain A; B, domain B; C, acidic C-tail), and (below) HMGB1 mutants and domains. HMGB1ΔC (amino acid residues 1–185). Positions of mutations are indicated. Data obtained with individual HMGB proteins are summarized on the *right*. oxi, oxidized. C22A/C44A, Cys at 22 and at 44 mutated to alanine. F37A, Phe at 37 mutated to alanine. #unpublished results. n.d., not determined.

**Fig 2:**
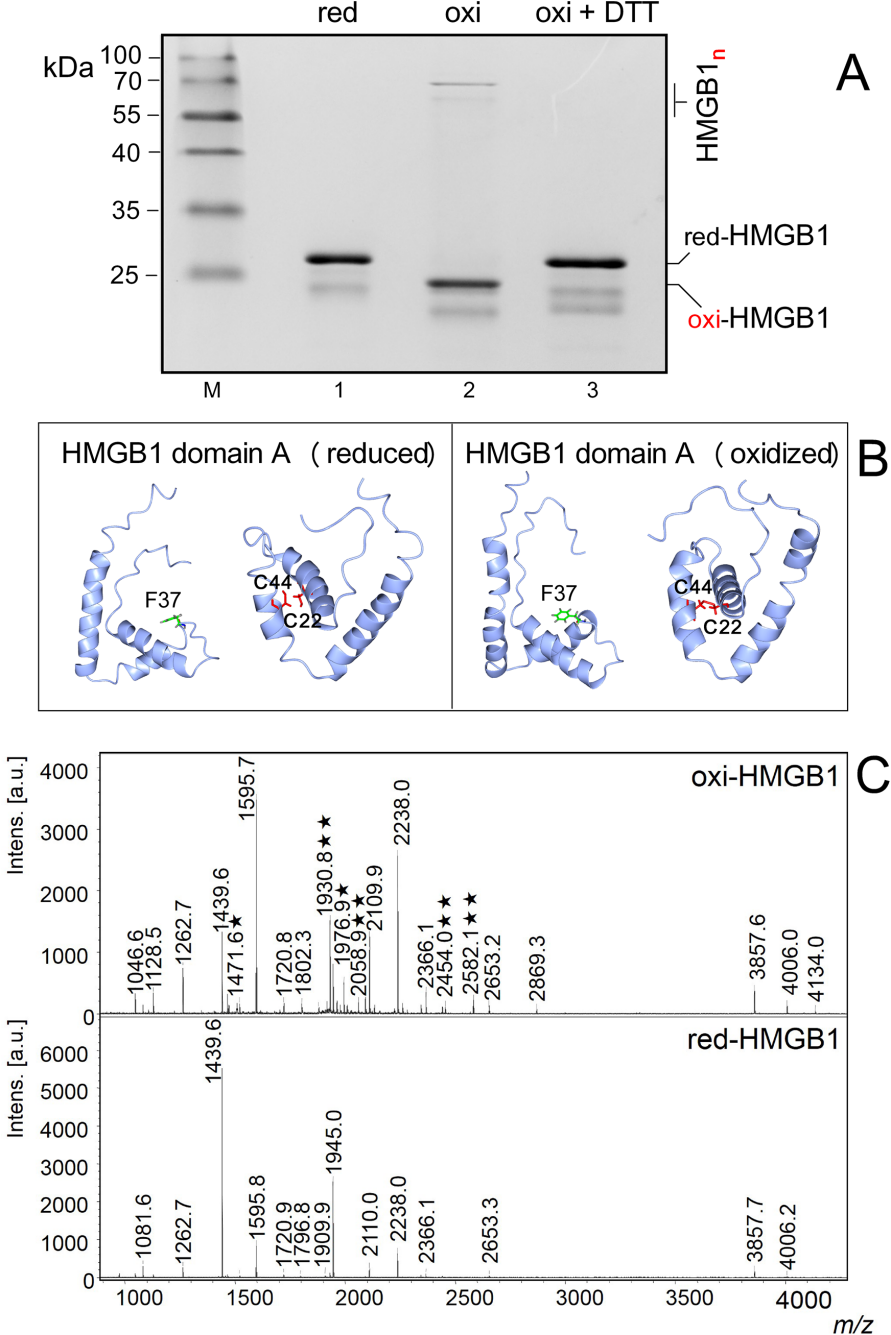
Oxidization of HMGB1 and analysis. **A**, polyacrylamide SDS gel electrophoresis of reduced HMGB1 (lane 1), mildly oxidized HMGB1 (lane 2, intramolecular cross-links) and oxidized HMGB1 treated with 10 mM DTT (lane 3). HMGB1n indicates position of intermolecular cross-links of HMGB1. M, molecular mass of the protein markers. **B**, comparison of published NMR structures of reduced HMGB1 domain A [23] and oxidized HMGB1 domain A [24]. CCP4MG program was used to draw the NMR structures using coordinates from the Protein Data Bank (1AAB and 2RTU for the reduced and oxidized form of domain A, respectively). **C**, comparison between MALDI-TOF mass spectra of tryptic digests of reduced and oxidized HMGB1. **, signals corresponding to peptides containing disulphide bridges between Cys22 and Cys44; *, signals corresponding to peptides oxidized to cysteine sulfoxide (< 5%). The identity of the oxidized HMGB1 peptides was proved by MALDI-MS/MS analysis. None of these signals (*data not shown*) were observed in the reduced HMGB1 protein (lane 1 in panel **A**) or after reduction of the oxidized HMGB1 protein by DTT (lane 3 in panel **A**), suggesting that mild-oxidization of HMGB1 was fully reversible.

HMGB1, HMGB1 mutants and truncated HMGB1 peptides were designed as outlined in Fig 1. HMGB1, domain A or HMGB1 lacking the acidic C-tail (referred as HMGB1ΔC) were subjected to mild oxidization by dialysis against low concentrations of Cu^2+^ and re-dialysis against buffer without Cu^2+^. As shown in Fig 2A (*lanes 1* and *2*), electrophoretic mobility of the oxidized HMGB1 was enhanced relative to the reduced form. The appearance of the faster moving band upon HMGB1 oxidation (Fig 2A, *lane 2*) was due to formation of an intramolecular disulphide bond by opposing Cys22 and Cys44 (Fig 2B), as confirmed by MALDI-TOF mass spectrometry of trypsin digested HMGB1 samples (Fig 2C). Interestingly, mild oxidization of HMGB1 also resulted in intermolecular cross-linking of several HMGB1 molecules (Fig 2A, *lane 2*). Oxidization of HMGB1 by Cu^2+^/O_2_ was fully reversible as (i) treatment of the oxidized HMGB1 protein with 10 mM DTT could reverse the electrophoretic mobility to that of the reduced protein (Fig 2A, compare *lanes 2* and *3*), and (ii) MALDI-TOF analysis confirmed the absence of a disulphide bond between Cys22 and Cys44 upon DTT treatment of mildly oxidized HMGB1 (not shown).

### The effect of oxidization and mutation of cysteines on preferential binding of HMGB1 to supercoiled DNA or DNA supercoiling

We have re-investigated the importance of cysteine residues of HMGB1 on the ability of the protein to bind or supercoil DNA in the presence of topoisomerase I. In our experiments we have used mild oxidization of HMGB1 or site-directed mutagenesis of Cys22/Cys44 of HMGB1, as opposed to modification of cysteine residues of HMGB1 by NEM (N-ethylmaleimide) used in previous report [10].

**Fig 3:**
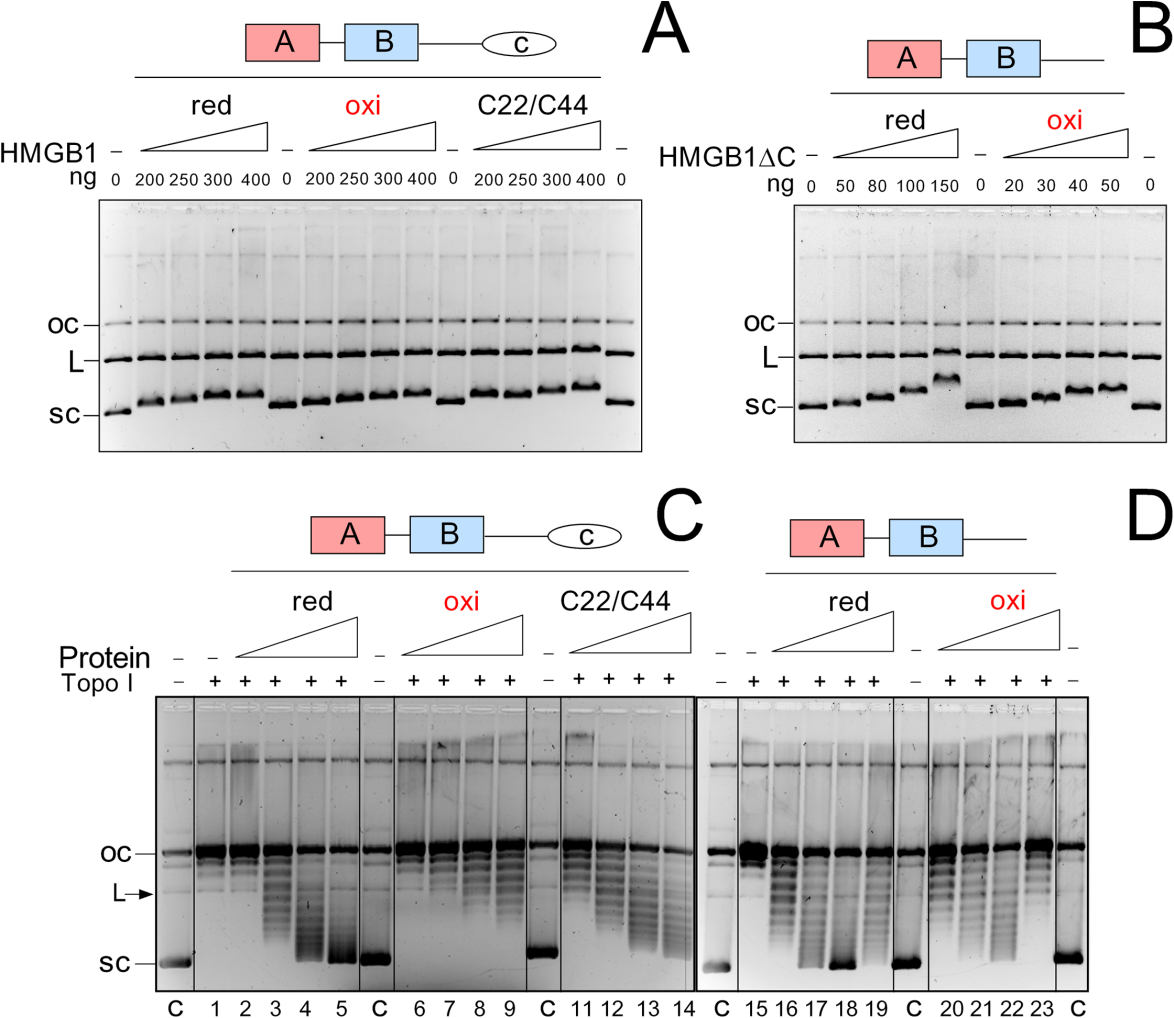
The effect of oxidization of HMGB1 on preferential binding to supercoiled DNA or DNA supercoiling. Panels **A** and **B**, preferential binding of HMGB1 or HMGB1 lacking the acidic C-tail (HMGB1ΔC) to supercoiled DNA. Proteins were pre-incubated with equimolar mixtures of supercoiled plasmid, relaxed circular plasmid and linearized plasmid pBR322, followed by resolution on 1% agarose gels. Notice that oxidization of HMGB1ΔC enhanced binding to supercoiled DNA compared to the reduced form of the protein, and therefore different amounts of reduced and oxidized HMGB1ΔC were used for the gel-retardation assay. Panels **C** and **D**, the effect of oxidization of HMGB1 on DNA supercoiling in the presence of topoisomerase I. Relaxed (circular) plasmid pBR322 DNA was incubated with increasing amount of HMGB1 or HMGB1ΔC at protein-to-DNA mass ratios of 1, 2, 4 and 6 (lanes 2–5,HMGB1-red; lanes 6–9, HMGB1-oxi; lanes 11–14, HMGB1 mutant; lanes 16–19, HMGB1ΔC-red; lanes 20–23, HMGB1ΔC-oxi) in the presence of topoisomerase I. Lanes C denote supercoiled DNA without addition of proteins. OC, relaxed, closed circular plasmid. L, linearized plasmid. SC, supercoiled plasmid. C22/C44, HMGB1(Cys22A/Cys44A) mutant. Deproteinised DNA samples were subjected to electrophoresis on 1% agarose gels, followed by staining with GelRed (Biotium).

First, we have studied the importance of cysteine residues of HMGB1 for binding to supercoiled DNA. Using gel retardation assay, we have found that the preferential binding of HMGB1 to supercoiled DNA was not significantly affected by mutation of Cys22/Cys44 or by formation of a disulphide bridge between Cys22 and Cys44 (Fig 3A). While oxidization of HMGB1 only slightly decreased retardation of supercoiled DNA relative to that of the reduced form of the protein, mutation of Cys22/Cys44 had no visible impact on binding to supercoiled DNA. Results of our DNA binding experiments with HMGB1(Cys22/Cys44) (Fig 3) or HMGB1 (Cys22/Cys44/Cys105) mutants (*unpublished results*) contradict to previously published data using NEM-modified HMGB1 indicating up to 90% inhibition of binding of the protein to supercoiled DNA [10], *see* the Discussion.

Similar binding experiments with HMGB1 lacking the acidic tail (HMGB1ΔC) revealed that the preferential binding of both reduced and oxidized HMGB1 to supercoiled DNA was not affected upon removal of the acidic C-tail (Fig 3B). Increased binding of reduced HMGB1ΔC to all types of ds-DNA was observed, in agreement with previous reports [10,11]. Oxidization of HMGB1ΔC further enhanced binding of the truncated HMGB1 protein to supercoiled DNA (Fig 3B), providing evidence that the involvement of cysteine residues of HMGB1 in DNA binding depends on the acidic C-tail of the protein.

We have also assessed the importance of cysteine residues of HMGB1 for DNA supercoiling. Previous report indicated that modification of cysteine residues of HMGB1 by NEM severely impaired the ability of the protein to supercoil DNA in the presence of topoisomerase I [10], suggesting a possible involvement of cysteines of HMGB1 in DNA supercoiling. However, the interpretation of the latter data could be likely affected both by modification of amino acid residues involved in DNA binding and direct inhibition of topoisomesase I by NEM. We have therefore re-investigated the involvement of cysteine residues of HMGB1 on the ability of the protein to supercoil closed circular DNA in the presence of topoisomerase I using either oxidized HMGB1 or HMGB1 mutated at Cys22/Cys44. As shown in Fig 3C, the number of supercoils increased with higher input HMG:DNA ratio. For the same molar input, the wild-type (reduced) HMGB1 protein was far more effective at DNA supercoiling than the oxidized protein. DNA supercoiling by reduced HMGB1(Cys22/Cys44) mutant was much less inhibited (Fig 3C), compared to experiments with NEM-treated HMGB1 (10) or oxidized HMGB1 (Fig 3C). Our experiments also demonstrated that the ability of HMGB1 to unwind DNA was markedly inhibited upon formation of an intramolecular disulphide bond by opposing Cys22 and Cys44 (due to oxidization), compared to mutation of the latter residues to alanine. Similar experiments with HMGB1ΔC revealed that the presence of the acidic C-tail could enhance the negative impact of oxidization on the ability of HMGB1 to supercoil DNA (Fig 3D).

### The effect of oxidization and mutation of cysteine residues of HMGB1 on DNA bending

Functioning of HMGB1 as an architectural protein in chromatin relies on the ability of the protein to induce DNA bending/looping [11], reviewed in [1,2,4]. Previously we have shown that mild oxidization of HMGB1could inhibit the ability of the protein to bend DNA [13]. Here, we made an attempt to elucidate the latter finding by comparing DNA bending by (reduced or oxidized) wild-type HMGB1 with DNA bending by two HMGB1 mutants: (i) HMGB1 (Cys22/Cys44 to alanine) and (ii) HMGB1 (Phe37 to alanine). HMGB1 mutant at Phe37 was selected from the following reasons. First, NMR studies revealed a flipped ring orientation of Phe37 in the oxidized form of HMGB1 domain A relative to that found in its reduced form [24], *see* also Fig 2B. Second, Phe37 was previously shown to be required for DNA bending due to intercalation into the DNA minor groove, reviewed in [4].

We have used DNA circularization assay to compare the DNA bending potential of HMGB1 or mutants. This assay measures the efficiency with which T4 DNA ligase forms circles from fragments of DNA below the persistence length (< 150-bp). In the absence of internal curvature, the stiffness of the short DNA fragments prevents intramolecular alignment of their ends so that circles are detected only in the presence of proteins that bend DNA [21,25–27], reviewed in [4].

**Fig 4:**
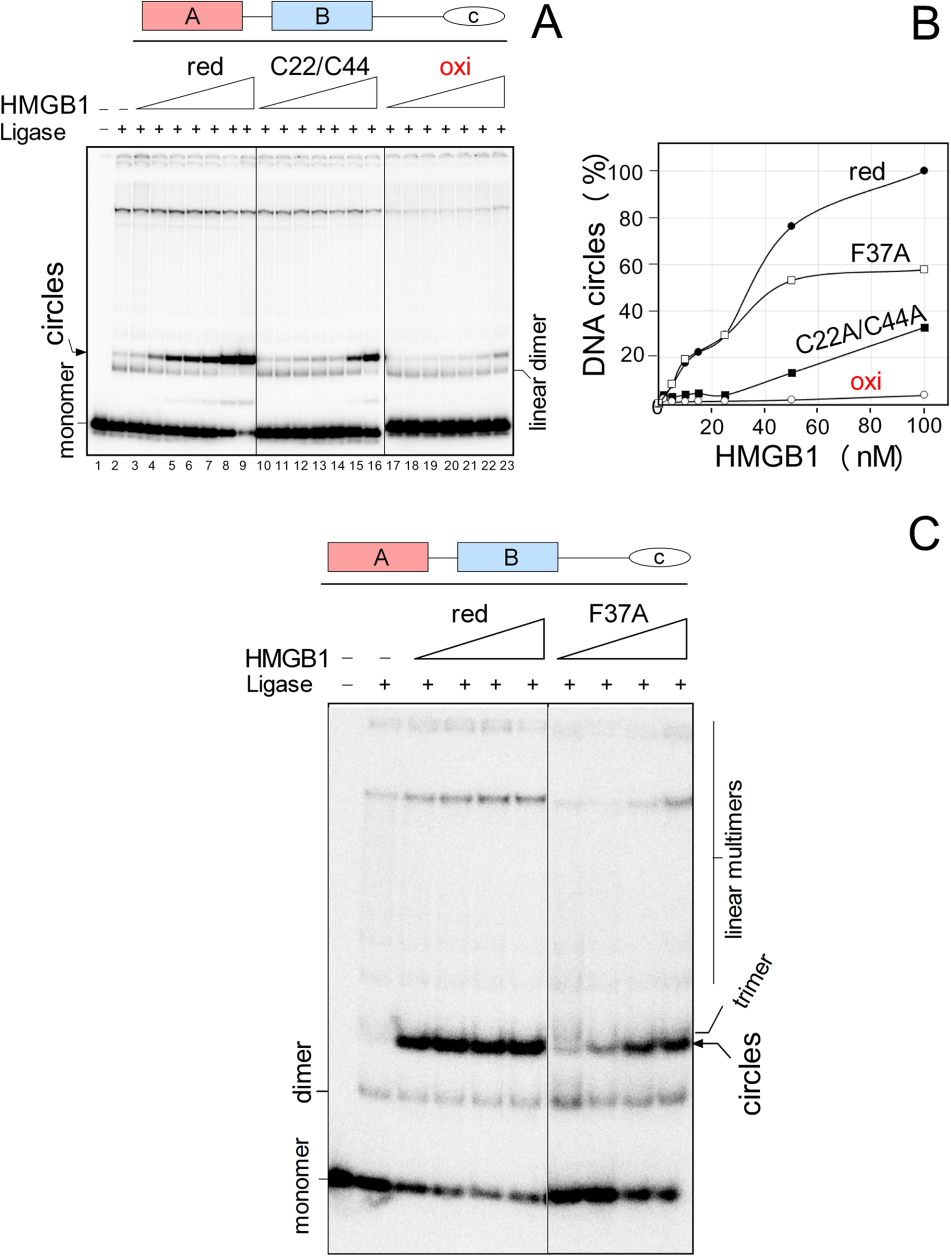
The effect of oxidization and mutation of Cys22/Cys44 or Phe37 of HMGB1 on DNA bending. **A**, the 5´-end ^32^P-labeled 123-bp DNA fragment (~1 nM) was preincubated with 2, 5, 10, 15, 25, 50 and 100 nM HMGB1 proteins (*left* to *right*), followed by ligation by T4 DNA ligase (DNA circularization assay). Deproteinised DNA samples were separated by electrophoresis on 5% non-denaturing polyacrylamide gels in 0.5x TBE buffer. **B**, percentage of DNA circles formed by reduced HMGB1, oxidized HMGB1 or HMGB1(Cys22A/Cys44A) mutant. The percentage of the minicircles formed at 100 nM HMGB1 was arbitrary set to 100% (each of the curves represent an average of three independent experiments). **C**, representative circularization assay using reduced HMGB1 and HMGB1(F37A) mutant (5, 20, 50 and 100 nM HMGB1, *left* to *right*). C22/C44, HMGB1(Cys22A/Cys44A) mutant.

As shown in Fig 4A, ligation of a 123-bp DNA fragment by T4 DNA ligase in the presence of increasing amounts of reduced HMGB1 resulted in a gradual appearance of DNA minicircles. The highest percentage of the minicircles was detected at 100 nM HMGB1 (arbitrary set to 100% in Fig 4B). Mutation of Cys22/Cys44 to alanine resulted in a moderate decrease in the percentage of minicircles (~30–40%), which was in contrast to oxidization of (wild-type) HMGB1, diminishing the percentage of DNA minicircles to only ~5–10% (Fig 4B). No DNA minicircles were observed with oxidized HMGB1 in the course of ligation of a 66-bp DNA duplex (*unpublished results*; a 66-bp DNA duplex is more difficult to bend than the 123-bp DNA duplex). There was a possibility that inhibition of DNA bending by oxidization of HMGB1 could be due to a flipped ring orientation of Phe37 in the oxidized HMGB1 domain A relative to that found in its reduced form [24], restricting intercalation of the residue into DNA, reviewed in [4]. In our next experiments, we have therefore studied the impact of replacement of Phe37 to alanine on DNA bending by HMGB1. As shown in Fig 4A, formation of DNA minicircles was visibly reduced at low concentrations of the HMGB1(Phe37A) mutant, and ~60% of the minicircles was detected at 100 nM of the HMGB1 mutant compared to those obtained with the wild-type HMGB1 (Fig 4B). Although DNA bending was inhibited both by oxidized HMGB1 and the HMGB1(Phe37A) mutant, the inhibition was more pronounced with the oxidized protein (Fig 4B). The latter differences cannot be solely explained by the absence of intercalation of Phe37 into DNA, and it is likely that other factors must contribute (*see* the Discussion). In addition, the fact that both oxidization and mutation of Phe37 to alanine could completely suppress the ability of the isolated HMGB1 domain A to bend DNA (S1 Fig)—unlike DNA bending by HMGB1(F37A) or oxidized HMGB1 (Fig 4)—suggested that the domain B within the full-length HMGB1 protein could partially compensate for the lack of the mutated or oxidized domain A to bend DNA.

**Fig 5:**
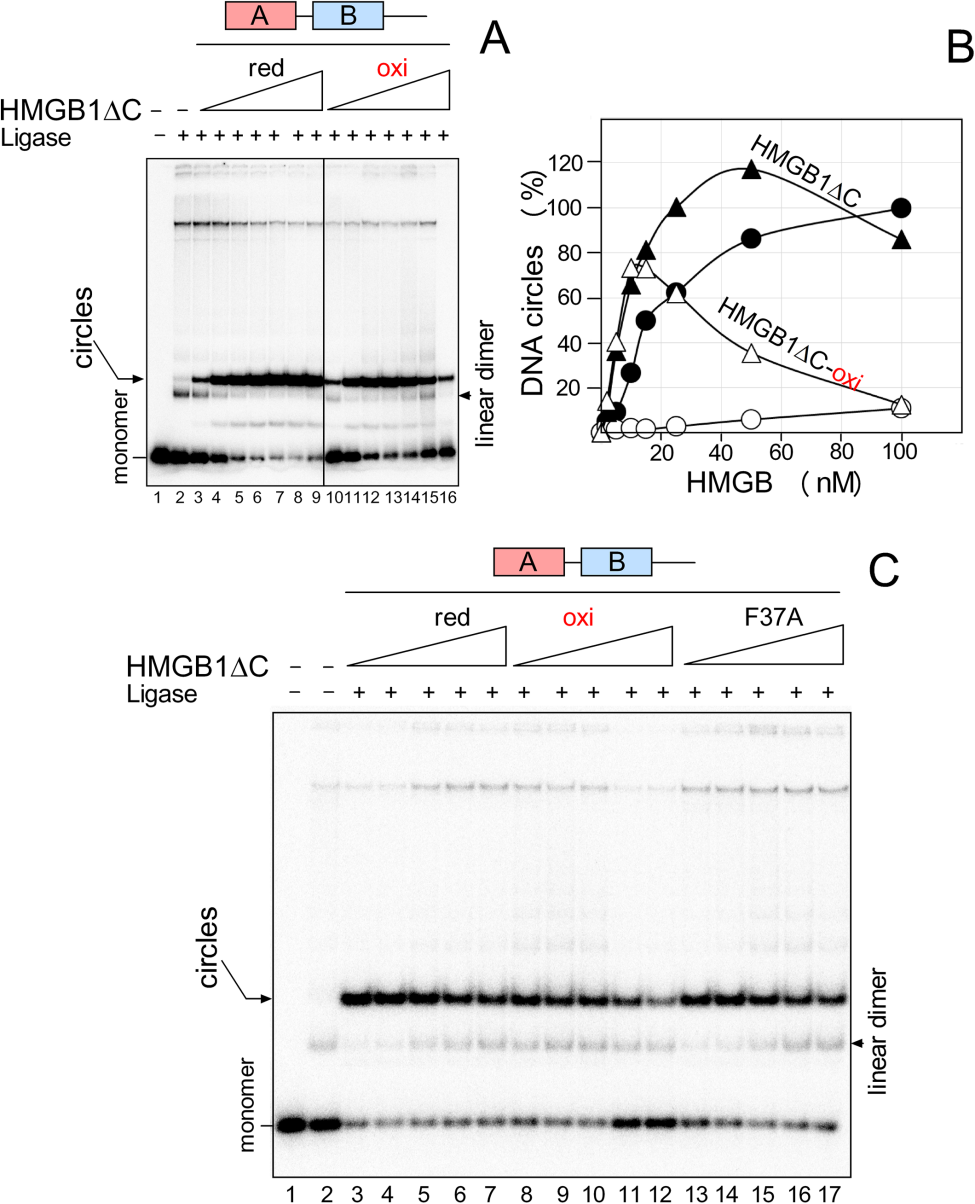
The effect of oxidization and mutation of Cys22/Cys44 or Phe37 of HMGB1ΔC on DNA bending. **A**, the 5´-end ^32^P-labeled 123-bp DNA fragment (~1 nM) was pre-incubated with 2, 5, 10, 15, 25, 50 and 100 nM of HMGB1 lacking the acidic C-tail (HMGB1ΔC, *left* to *right*), followed by ligation by T4 DNA ligase (DNA circularization assay). Deproteinised DNA samples were separated by electrophoresis on 5% non-denaturing polyacrylamide gels in 0.5x TBE buffer. **B**, percentage of DNA circles formed by reduced (black triangle) or oxidized (empty triangle) HMGB1ΔC, as compared to DNA circles formed under the same conditions by reduced (black circles) or oxidized (empty circles) full-length HMGB1. The percentage of the minicircles formed at 100 nM HMGB1 was arbitrary set to 100% (each of the curves represent an average of three independent experiments). **C**, representative circularization assay using reduced HMGB1ΔC, oxidized HMGB1ΔC, and HMGB1ΔC(F37A). Concentrations of proteins were 5, 10, 25, 50 and 100 nM (*left* to *right*).

Previously it has been reported that DNA binding properties of the HMG boxes are modulated by the acidic C-tail of HMGB1 [10,11], reviewed in [4,28]. We have therefore studied the possible importance of the acidic C-tail, and the impact of the oxidization, on DNA bending. As shown in Fig 5B, HMGB1ΔC was slightly more efficient in formation of DNA minicircles than the full-length HMGB1 protein at the same molar concentrations, in agreement with previous reports [21,27]. Oxidization of HMGB1ΔC had very little, if any effect, on formation of minicircles until ~20 nM concentration of the peptide (Fig 5B). However, a gradual decrease in formation of minicircles was observed at >20 nM of the oxidized peptide, until the percentage of DNA minicircles was at 100 nM HMGB1ΔC similar to that observed with the oxidized full-length HMGB1 (~5–10%). Mutation of Phe37 affected the ability of HMGB1 to bend DNA much less than the oxidization of the protein, regardless the presence or absence of the acidic C-tail (compare Figs 4A and 5C).

The main conclusion from our DNA bending experiments is: (i) the ability of HMGB1 to bend DNA is severely compromised upon oxidization or mutation of Phe37 to alanine, whereas (ii) a double mutation of Cys22 and Cys44 to alanine has a much lower impact on DNA bending, compared to formation of a disulphide bond between Cys22 and Cys44 upon oxidization of the protein. The above findings on DNA bending are in agreement with our data on the impact of oxidization/mutation of Cys22/Cys44 (Fig 3) and mutation of Phe37 (*unpublished results*) on DNA unwinding. We conclude that the negative impact of oxidization or mutation of Phe37) on the ability of HMGB1 to bend or unwind DNA is enhanced by the acidic C-tail.

### Modulation of HMGB1-mediated DNA bending by histone H1

The acidic C-tail can modulate interactions of the HMG-boxes of HMGB1 with a plethora of cellular proteins, and in some cases, the tail can directly interact with proteins, reviewed in [4,29]. Several factors have been shown to affect the interactions of the HMG-boxes with DNA or other proteins, presumably via competing off the acidic C-tail of HMGB1 [13,15,17,18]. These factors include linker histones (H1 and H5) which can interact with the acidic C-tail of HMGB1 [17,18], reviewed in [4,15].

**Fig 6:**
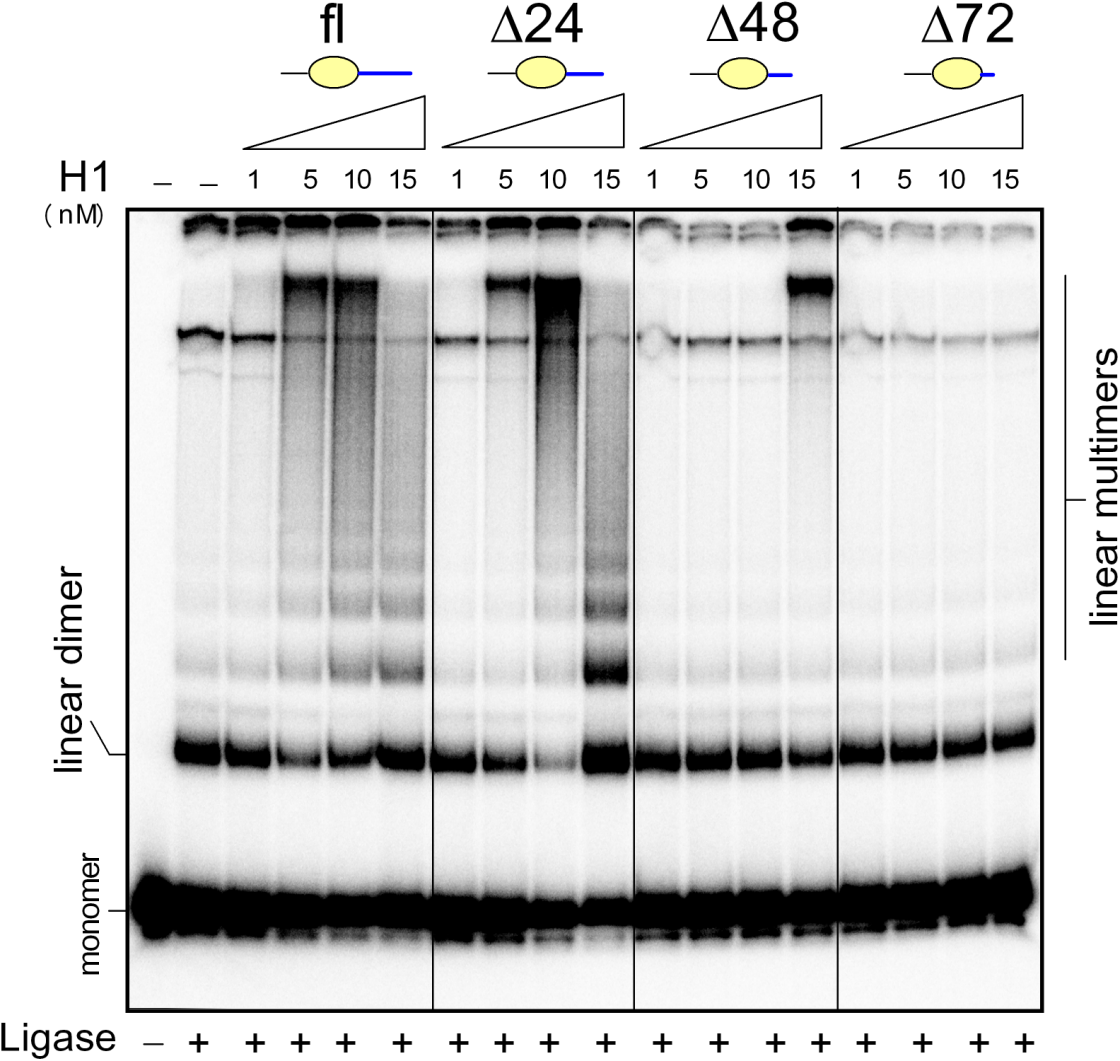
Stimulation of DNA ligation by histone H1 and deletion mutants. The 5´-end ^32^P-labeled 123-bp DNA fragment (~1 nM) was pre-incubated with 1–15 nM (*left* to *right*) histone H1 (fl) or deletion mutants within the highly basic C-terminus, followed by ligation by T4 DNA ligase. Deproteinised DNA samples were separated by electrophoresis on 5% non-denaturing polyacrylamide gels in 0.5x TBE buffer.

In this paper we have studied a possible impact of histone H1 on the ability of HMGB1 of different redox state to bend DNA (DNA circularization assay). First, we have performed control experiments to ascertain the effect of histone H1 on ligation of a 123-bp DNA duplex in the absence of HMGB1. In agreement with previous reports [30,31], histone H1 could stimulate formation of linear multimers by T4 DNA ligase at low H1-to-DNA ratios. The highest stimulation of DNA ligation was observed at 5–10 nM H1 (Fig 6), whereas at >25 nM H1 inhibition of DNA ligation was detected (*not shown*), suggesting the existence of a narrow concentration optimum for stimulation of DNA joining by histone H1. Removal of 24 amino acids from the very C-terminus of H1 (H1Δ24) had very little, if any, effect on the stimulatory effect of the H1 peptide on DNA ligation. Further deletion of the C-terminus of H1 resulted in the inability of the truncated H1 peptides (H1Δ48 or H1Δ72, Fig 6, and H1Δ97, S2 Fig) to stimulate DNA ligation. We have confirmed that under the conditions of our experiments, no DNA minicircles were detected in the course of DNA ligation at any histone H1-to-DNA ratio studied (Fig 6).

**Fig 7:**
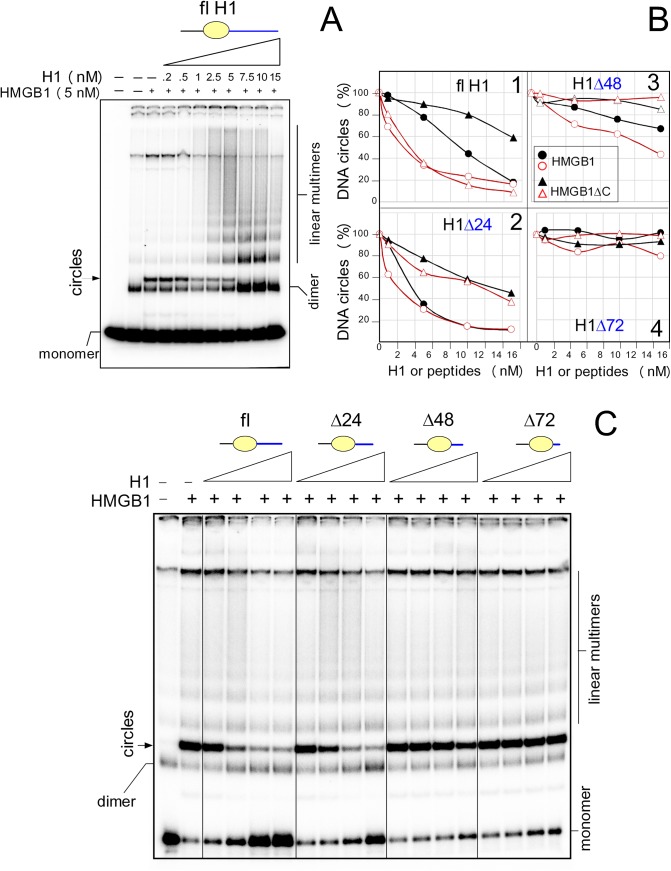
Histone H1 inhibits the ability of HMGB1 to bend DNA. **A**, formation of DNA circles by HMGB1 is inhibited by the full-length histone H1 (DNA circularization assay). The 5´-end ^32^P-labeled 123-bp DNA fragment (~1 nM) was pre-incubated with 5 nM HMGB1, followed by titration with increasing concentrations of H1 (0.2–15 nM, *left* to *right*) and ligation by T4 DNA ligase. Deproteinised DNA samples were separated by electrophoresis on 5% non-denaturing polyacrylamide gels in 0.5x TBE buffer. Panels **B**-**E**, DNA circularization assays in the presence of the full-length histone H1(fl) or peptides H1Δ24, H1Δ48 and H1Δ72. The percentage of DNA circles by reduced or oxidized HMGB1 or HMGB1ΔC (50 nM) in the presence of increasing concentrations of H1 or H1 peptides (1–15 nM, *left* to *right*) is indicated. The percentage of the minicircles formed by HMGB1 or HMGB1ΔC in the absence of H1 or peptides was arbitrary set to 100%. Oxidized HMGB1 or HMGB1ΔC proteins are indicated in red.

Next, we have investigated the influence of low concentrations of histone H1 on DNA bending by HMGB1. As shown in Fig 7A, formation of DNA minicircles by HMGB1 was ~2-3-fold decreased already at an H1-to-HMGB1 molar ratio of 0.2, and at H1-to-HMGB1 molar ratios > 1, very little, if any, DNA minicircles were observed. Our data provided evidence that H1 could inhibit the ability of HMGB1 to bend DNA. Due to low efficiency of oxidized HMGB1 or HMGB1ΔC in promotion of formation of DNA minicircles, further circularization assays were performed at 10-times higher concentrations of HMGB1 or HMGB1ΔC than in Fig 7A. A typical experiment on the impact of histone H1 or H1 peptides of different lengths of the highly basic C-termini on the HMGB1-mediated DNA bending is shown in Fig 7C. Similar experiments were also performed with oxidized HMGB1, as well as with reduced and oxidized HMGB1ΔC, and the obtained data are summarized in Fig 7B (each of the curves represents an average of three independent experiments). As shown in Fig 7B (panel 1), histone H1 was the most efficient in inhibition of DNA bending by the full-length HMGB1 (~90% inhibition at 15 nM H1; higher concentrations of H1 could not be studied due to the inhibitory effect of H1 on DNA ligation reaction, in agreement with previous reports [31,32]. We have observed that oxidization of HMGB1 markedly enhanced the inhibitory effect of histone H1 on DNA bending by HMGB1 (Fig 7B, pane 1). Interestingly, histone H1 could also partially inhibit DNA bending by HMGB1ΔC (~40% inhibition, Fig 7B, pane 1), and the inhibition was enhanced by oxidization of HMGB1ΔC. These data provided evidence that although histone H1 could inhibit DNA bending by both HMGB1 and HMGB1ΔC, the inhibition was more efficient if HMGB1 contained the acidic C-tail.

**Fig 8:**
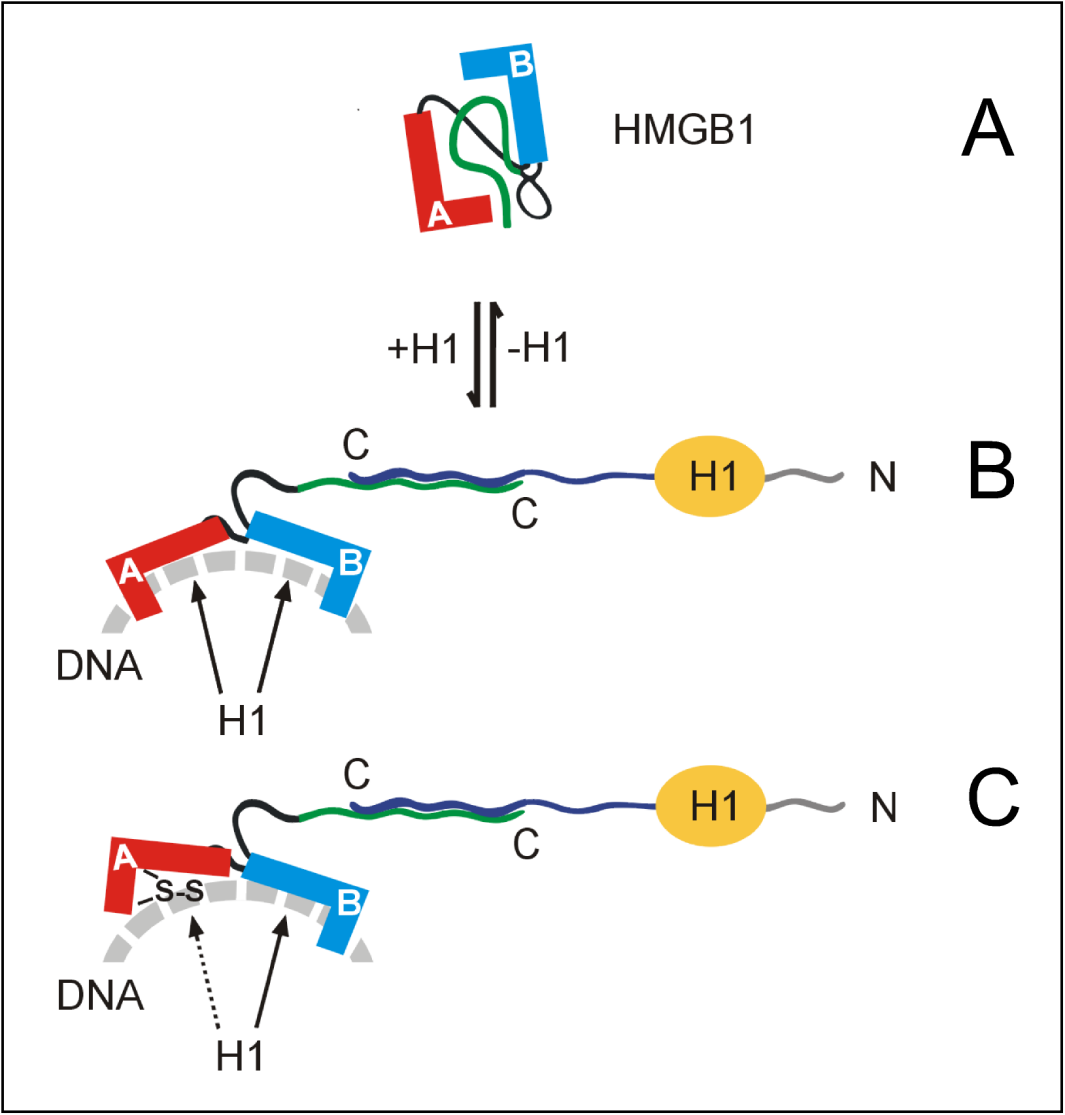
H1-HMGB1 interplay and possible impact on DNA bending by HMGB1. **A**, folded structure of HMGB1 protein showing the acidic C-tail (green) in contact with the HMG-boxes. Binding of a region encompassing ~ 48 amino acids of the very end of the highly basic C-terminus of histone H1 (dark blue) to the acidic C-tail of HMGB1 (green) can release the HMG-boxes A+B for DNA binding/bending (panels **B** and **C**). Histone H1 can also weaken HMGB1 binding to DNA by direct interaction with the HMG-boxes (arrows) and/or displacement of the HMG-boxes by virtue of higher affinity of H1 for DNA (panel **B**). The inhibitory effect of H1 on DNA bending by HMGB1 is enhanced by oxidization of HMGB1. Oxidization of HMGB1 results in lower DNA binding/bending of the HMG-box A (red, a disulphide bond is indicated), promoting displacement of both HMG-boxes from DNA by histone H1 (dashed and solid arrows in panel **C**). Adapted from Ref. [18].

Previous reports demonstrated that histone H1 could interact with the acidic C-tail of HMGB1 via its highly basic C-terminus [18], reviewed in [15]. In order to clarify which part of the C-terminus of H1 is involved in the observed inhibition of DNA bending, similar circularization assay were performed with H1 deletion mutants within the basic C-terminus. As shown in Fig 7B (panel 2), removal of 24 amino acids from the very C-terminus of H1 (H1Δ24 peptide) could slightly enhanced the inhibitory effect of histone H1 on DNA bending by reduced HMGB1 or HMGB1ΔC. Interestingly, no differences in DNA bending by the reduced and oxidized forms of HMGB1 or HMGB1ΔC were observed in the presence of H1Δ24 peptide (Fig 7B, panel 2). Removal of 48 amino acids from the very C-terminus of H1 resulted in H1 peptide (H1Δ48) which could no longer inhibit DNA bending by HMGB1ΔC (Fig 7B, panel 3), and only slightly DNA bending by HMGB1. No inhibition of either the HMGB1- or HMGB1ΔC-mediated DNA bending (regardless the redox state of the proteins) was observed with histone H1 lacking 72 amino acids from the very end of the C-terminus (H1Δ97 peptide, Fig 7B, panel 4). Similar results were obtained using H1Δ97 peptide, corresponding to H1 lacking the highly basic C-terminus (S2 Fig). In summary, our results demonstrated that a region encompassing ~48 amino acid residues from the very C-terminus of the highly basic C-terminal domain of H1 is required for a strong inhibition of HMGB1-mediated DNA bending. The extent of the inhibition was further dependent on the presence of the acidic C-tail and the redox state of HMGB1 (Fig 8, *see* the Discussion).

## Discussion

HMGB1 protein is a member of the High Mobility Group (HMG) superfamily, acting as a DNA chaperone by DNA unwinding and bending/looping, influencing multiple processes in chromatin [4]. Many of the intracellular and extracellular functions of HMGB1 depend on redox-sensitive cysteine residues of the protein, reviewed in [7]. Here, we report that formation of a disulphide bridge between Cys22 and Cys44 by mild oxidization of HMGB1, and much less mutation of the cysteine residues, severely compromised the functioning of the protein as a DNA chaperone by inhibiting its ability to supercoil DNA in the presence of topoisomerase I. HMGB1 modified with cysteine-specific reagent NEM [10], unlike HMGB1(Cys22A/Cys44A) (this report) or HMGB1(Cys22/Cys44A/Cys105A) (*unpublished*), could markedly inhibit the ability of HMGB1 to bind or supercoil DNA. We believe, that the discrepancy may be due to the fact that treatment of HMGB1 with NEM could result (in addition to specific modification of cysteines) in modification of other residues involved in DNA binding and inhibition of topoisomerase I. Our experiments provided evidence that the involvement of cysteine residues of HMGB1 in DNA binding depends on the acidic C-tail, enhancing the negative impact of oxidization on the ability of HMGB1 to unwind DNA.

Functioning of HMGB1 in chromatin also relies on the ability of the protein to induce DNA bending/looping ([11], reviewed in [4]). DNA bending by HMGB1 requires intercalation of residues Phe37 (domain A) and Phe102/Ile112 (domain B) into the DNA minor groove of DNA (reviewed in [4]). Domain B, and much less domain A, is responsible for DNA bending *in vitro* [21,33,34]. This can explain why oxidized HMGB1 or HMGB1(F37A) mutant could still bend DNA despite the complete loss of the ability of oxidized or mutated isolated domain A to bend DNA (this report). The observed inhibition of DNA bending by oxidized HMGB1 may be due to a flipped ring orientation of Phe37 in the oxidized domain A as revealed by NMR [24], restricting intercalation of Phe37 into DNA. The importance of Phe37 for bending of linear DNA was recently supported from the crystal structure of HMGB1 domain A bound to AT-rich DNA fragment, indicating stacking of two Phe37 residues of both domains A together and intercalation of the same CG base pair [35]. Although DNA bending was significantly compromised both by oxidized HMGB1 and HMGB1(Phe37) mutant (this paper), the inhibition was more apparent with the oxidized protein. Our data indicate that, in addition to impaired intercalation of Phe37 into DNA, other factor(s) could account for the observed inhibition of DNA bending by the oxidized HMGB1, such as an oxidation-induced conformational change of HMGB1. However, in the absence of any published structure of HMGB1, we can only speculate that the reported subtle conformational change of the oxidized domain A [24] is able to affect the global structure of HMGB1. In indirect support of the latter idea, a double mutation of Cys22/Cys44 to alanine in HMGB1 (which is not expected to induce any major conformational change in the protein) had much lower impact on DNA bending or unwinding than formation of a disulphide bond between Cys22 and Cys44. Our experiments further revealed that the acidic C-tail could markedly enhance the negative impact of oxidization or mutation of Phe37 on the ability of HMGB1 to bend or unwind DNA, suggesting that the acidic C-tail is an important factor modulating the involvement of cysteine residues of HMGB1 in DNA bending or unwinding.

One of the consequences of functioning of HMGB1 as a DNA chaperone is promotion of chromatin re-modelling and enhancement specific binding of transcription factors to DNA, reviewed in [4]. Although the precise sequence of events in HMGB1-mediated augmentation of binding of transcription factors to DNA is unclear, the presumed mechanisms involve direct protein-HMGB1 interactions helping to deliver specific proteins to their cognate sites and/or DNA bending and unwinding by HMGB1. HMGB1, like histone H1, binds to linker DNA in chromatin, suggesting that their binding may be mutually exclusive [18]. This could also account for a population of mononucleosomes with stoichiometric amounts of HMGB1, but depleted with H1 [36]. Although the data about the available nuclear pools of HMGB1 and histone H1, and their physical localization that could facilitate their mutual competition, are still missing [37], photobleaching techniques revealed that HMGB1 can move rapidly throughout the entire nucleus, reflecting the transient binding of HMGB1 [38]. HMGB1 is the most mobile chromatin-associated protein and only 1–2 seconds is required to cross the nucleus [39]. On the other hand, histone H1 is much less mobile protein in the nucleus, with an average binding time of each H1 molecule on chromatin being ~4 min [40]. Interaction of HMGB1 with histone H1 could efficiently decrease mobility of HMGB1 (as well as H1), which could subsequently limit the ability of HMGB1 to bend DNA and to exercise its role as an architectural factor in chromatin.

In an effort to understand one of the aspects of a possible interplay between histone H1 and HMGB1, we have studied whether histone H1 could modulate the DNA bending potential of HMGB1 *in vitro*. We report for the first time that histone H1 can significantly compromise the ability of HMGB1 to bend DNA. We have shown that the region encompassing ~48 amino acids from the very C-terminus of histone H1 is required for a strong inhibition of DNA bending by HMGB1. Histone H1 was the most efficient in inhibition of DNA bending by the full-length HMGB1, compared to the HMGB1ΔC, consistent with the reported requirement of the acidic C-tail of HMGB1 for binding to the highly basic C-terminus of histone H1 [17,18], reviewed in [4,15]. We have also demonstrated that DNA bending by oxidized HMGB1, and much less by the reduced protein, was more susceptible to inhibition by histone H1. The latter finding cannot be explained as a (solely) consequence of H1 binding to HMGB1 as tryptophan fluorescence quenching [16], chemical cross-linking [13] or Micro Scale Thermophoresis (*unpublished results*) revealed significantly lower affinity of H1 for oxidized HMGB1, compared to the reduced form of the protein. We believe that the inhibition of HMGB1-mediated DNA bending by histone H1 may be a consequence of a partial or full displacement of HMGB1 from DNA by histone H1, which is facilitated by direct H1-HMGB1 interactions. This idea is in agreement with *in vitro* studies demonstrating significantly higher affinity of histone H1 for linear DNA compared to HMGB1 [41], enabling replacement of HMGB1 by histone H1. Lower affinity of oxidized HMGB1 for DNA [12,13] can further enhance the inhibitory effect of histone H1 on DNA bending. While efficient inhibition of reduced HMGB1-mediated DNA bending by histone H1 strongly depends on the acidic C-tail of HMGB1, histone H1 can inhibit DNA bending by oxidized HMGB1 or HMGB1ΔC with a comparable efficiency. Our finding that histone H1 can partially inhibit DNA bending by reduced or oxidized HMGB1 lacking the acidic C-tail (albeit with a distinct efficiency) may suggest that other region(s) in HMGB1 (*not* the acidic C-tail) are also involved in the H1-mediated inhibition of DNA bending. This idea is indirectly supported by our Micro Scale Thermophoresis measurements indicating a weak binding of H1 to HMGB1ΔC (*unpublished results*). It is possible that displacement (or weakening of DNA binding) of HMGB1 or HMGB1ΔC from DNA by histone H1 can represent a common mechanism by which H1 inhibits DNA bending by the proteins. Binding of the end region of the highly basic C-terminus of H1 to the acidic C-tail of HMGB1 and/or DNA can facilitate the displacement (weakening?), resulting in decreased DNA bending by HMGB1. Oxidative stress could further promote the inhibitory role of histone H1 on HMGB1-mediated DNA bending (Fig 8). However, it is unclear to what extent and in what order the H1-HMGB1 interactions and/or H1-mediated displacement of HMGB1 contribute to the observed inhibition of DNA bending by histone H1.

In summary, we have shown that histone H1 can inhibit DNA bending by HMGB1 *in vitro*. The extent of the inhibition depends on the presence of the acidic C-tail and/or redox state of HMGB1. The observed inhibitory effect of H1 on DNA bending by HMGB1 could have significant consequences for the functioning of HMGB1 as a DNA chaperone in chromatin. Replacement of HMGB1 by linker histones H1 (or vice versa, [13] and Refs. therein) from their binding sites in nucleosomes could bring about changes in chromatin stability, associated with distinct recruitment of biologically important proteins with different impact on transcriptional activation.

## Supporting Information

S1 Fig Oxidization or mutation of Phe37 inhibits DNA bending by the HMGB1 domain A. DNA circularization assay. ^32^P-labeled 123-bp DNA duplex was ligated with T4 DNA ligase in the presence of HMGB1 domain A (0.5, 1.5, 3 and 4.5 μM, *left to right*). Deproteinised DNA samples were resolved on 5% polyacrylamide gels in 0.5 x TBE. red, reduced HMGB1 domain A. oxi, oxidized HMGB1 domain A. F37A, HMGB1 domain A mutated at Phe37 to alanine. Oxidized and reduced domains A in the *left* panel represent untagged proteins. Reduced domain A and the F37A mutant in the *right* panel represent His-tagged proteins.
(TIF)

S2 Fig Histone H1 lacking the highly basic C-terminus cannot inhibit DNA bending by HMGB1. Formation of DNA circles by HMGB1 is inhibited by the full-length histone H1 (DNA circularization assay). The 5´-end ^32^P-labeled 123-bp DNA fragment (~1 nM) was pre-incubated with 50 nM HMGB1, followed by titration with increasing concentrations of H1 or H1 lacking the basic C-terminus (H1Δ97 peptide) (1, 5, 10, 15 and 25 nM, *left to right*) and ligation by T4 DNA ligase. Deproteinised DNA samples were separated by electrophoresis on 5% non-denaturing polyacrylamide gels in 0.5x TBE buffer.
(TIF)

## Abbreviations

HMG: High Mobility Group
HMGB1-red: reduced HMGB1 protein
HMGB1-oxi: oxidized HMGB1 protein
CTD: C-terminal Domain
EMSA: Electrophoresis Mobility Shift Assay
PAGE: Polyacrylamide Gel Electrophoresis
NEM: N-ethylmaleimide
