# Supplementary figures and images for: Histone H1 Differentially Inhibits DNA Bending by Reduced and Oxidized HMGB1 Protein
Source: PLoS One. 2015 Sep 25;10(9):e0138774. doi: 10.1371/journal.pone.0138774 (PMC4583294; doi:10.1371/journal.pone.0138774)

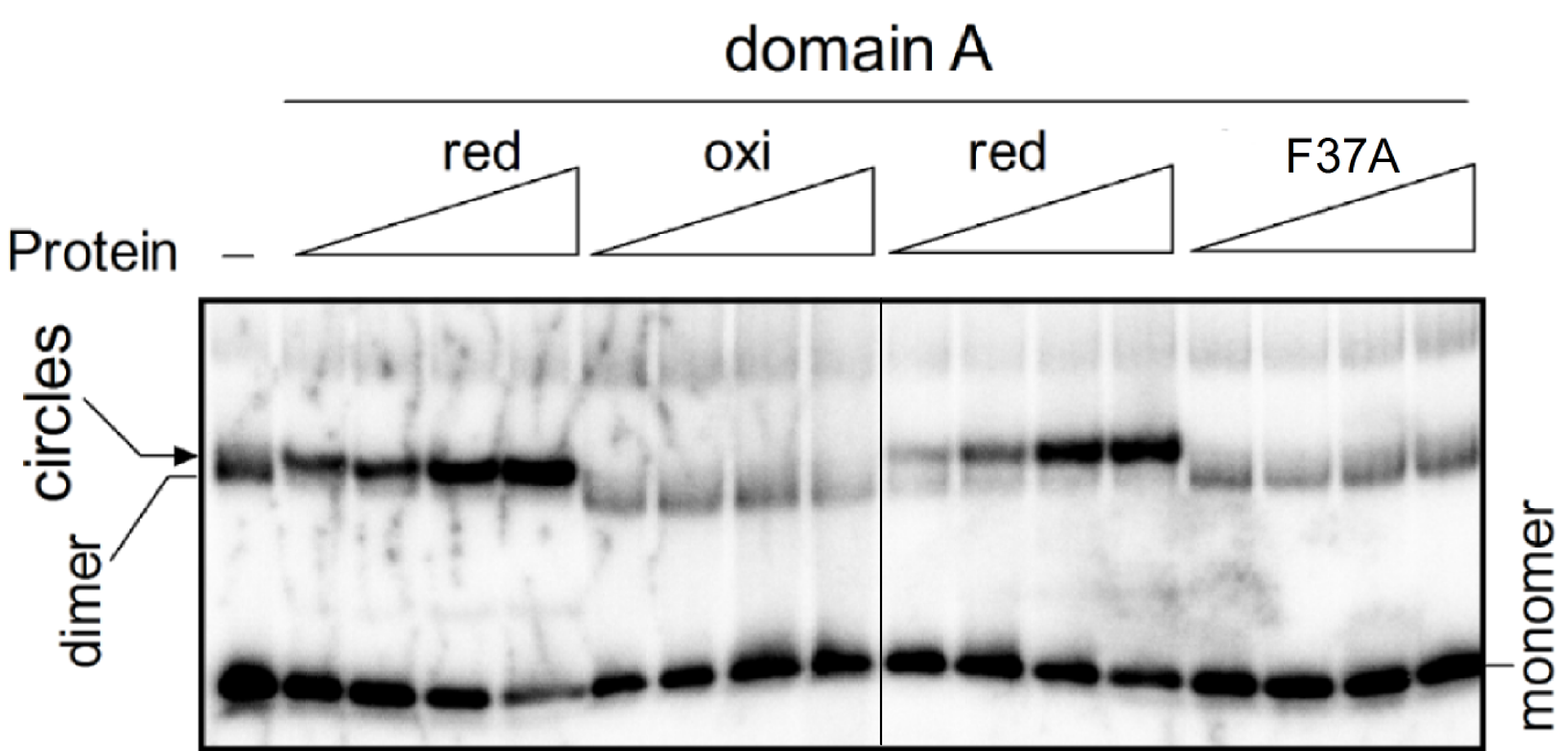

Supplement: S1 Fig — DNA circularization assay. 32P-labeled 123-bp DNA duplex was ligated with T4 DNA ligase in the presence of HMGB1 domain A (0.5, 1.5, 3 and 4.5 μM, left to right). Deproteinised DNA samples were resolved on 5% polyacrylamide gels in 0.5 x TBE. red, reduced HMGB1 domain A. oxi, oxidized HMGB1 domain A. F37A, HMGB1 domain A mutated at Phe37 to alanine. Oxidized and reduced domains A in the left panel represent untagged proteins. Reduced domain A and the F37A mutant in the right panel represent His-tagged proteins. (TIF) [file pone.0138774.s001.tif]

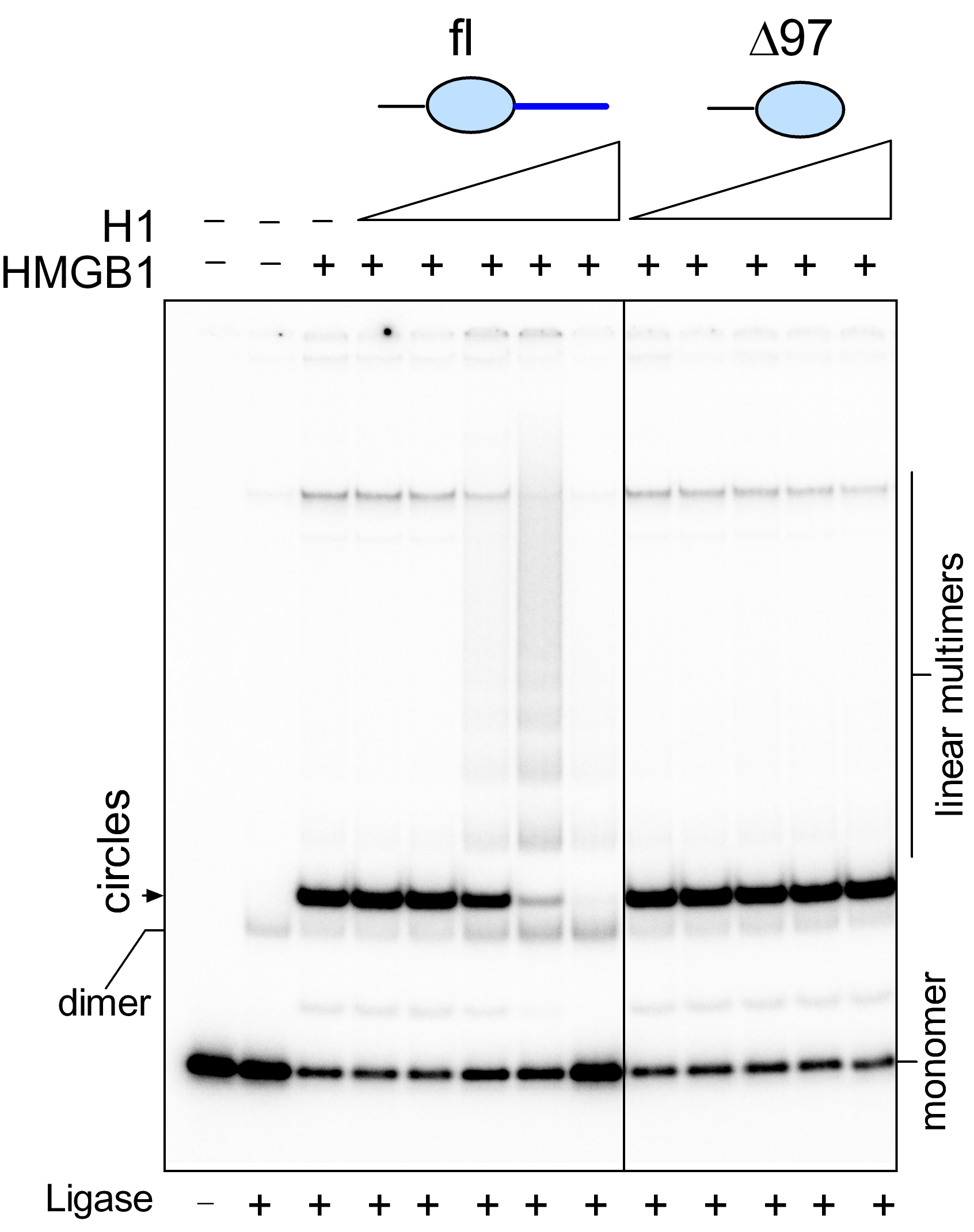

Supplement: S2 Fig — Formation of DNA circles by HMGB1 is inhibited by the full-length histone H1 (DNA circularization assay). The 5´-end 32P-labeled 123-bp DNA fragment (~1 nM) was pre-incubated with 50 nM HMGB1, followed by titration with increasing concentrations of H1 or H1 lacking the basic C-terminus (H1Δ97 peptide) (1, 5, 10, 15 and 25 nM, left to right) and ligation by T4 DNA ligase. Deproteinised DNA samples were separated by electrophoresis on 5% non-denaturing polyacrylamide gels in 0.5x TBE buffer. (TIF) [file pone.0138774.s002.tif]
